# Uncontrolled Hypertension Increases with Age in an Older Community-Dwelling Chinese Population in Shanghai

**DOI:** 10.14336/AD.2016.1220

**Published:** 2017-10-01

**Authors:** Sheng Peng, Ting Shen, Jie Liu, Brian Tomlinson, Huimin Sun, Xiaoli Chen, Paul Chan, YaShu Kuang, Liang Zheng, Hong Wu, Xugang Ding, Dingguang Qian, Yixin Shen, Pingjin Gao, Huimin Fan, Zhongmin Liu, Yuzhen Zhang

**Affiliations:** ^1^Key Laboratory of Arrhythmias, Ministry of Education, Research Center for Translational Medicine, Shanghai East Hospital, Tongji University School of Medicine, Shanghai 200120, China; ^2^Department of Medicine and Therapeutics, The Chinese University of Hong Kong, Hong Kong SAR, China; ^3^Division of Cardiology, Department of Internal Medicine, Wan Fang Hospital, Taipei Medical University, Taipei, Taiwan; ^4^Gaohang Community Medical Center, Shanghai, 201208, China; ^5^Shanghai Hypertension Institute, Rui Jin Hospital, JiaoTong Univeristy School of Medicine, Shanghai, 200120, China

**Keywords:** hypertension, prevalence, cardiovascular disease, older Chinese community population

## Abstract

We determined the prevalence of hypertension, medication usage and attainment of blood pressure goals in older (≥65 to <80 years and ≥80 years) urban community-dwelling Chinese subjects. Data were obtained in 3950 subjects (mean age 72.0 years, 1745 male) including 609 subjects aged ≥80 years in the Shanghai Elderly Cardiovascular Health Study (SHECHS). Established cardiovascular disease was present in 7.7% of participants. The prevalence of hypertension was 74.8% overall and it was more than 80% in individuals considered to be in moderate and higher cardiovascular disease risk categories. In hypertensive subjects, 67.1% were on treatment and treatment was more frequent in high and very high cardiovascular risk individuals. Attainment of the systolic blood pressure goal <150 mmHg was 62.9% and was greater in the ≥65 to <80 years group than in the ≥80 years group. The most commonly used antihypertensive treatments were calcium channel blockers (54.2%), followed by angiotensin receptor blockers (43.1%). Diuretics were used in 2.6%. Fixed-dose combination antihypertensive tablets were used in some of the ≥65 to <80 years group (12.4%) and more of the ≥80 years group (18.2%) and 70.9% of the ≥65 to <80 years group and 80.2% of the ≥80 years group were on monotherapy. There were high prevalence and high treatment rates of hypertension, but poor attainment of the systolic blood pressure goal of <150 mmHg, especially in the ≥80 years group of community-dwelling Chinese. Considering that more intensive treatment of hypertension in older subjects may be warranted after recent studies, this might be achieved by more frequent use of combinations of effective therapies and diuretics.

Hypertension is the leading risk factor for cardiovascular disease (CVD) mortality worldwide [1]. Whilst blood pressure (BP) levels have decreased in the Japanese population over the past 50 years [2], hypertension prevalence rates in China have increased significantly and were 5.1%, 7.7%, 13.6% and 18.8% in 1959, 1979-1980, 1991 and 2002, respectively [3]. In 2006 to 2008, the prevalence of hypertension in subjects ≥60 years in Shanghai was >59% [4]. With the acceleration of population aging, older people ≥80 years are estimated to be the most rapidly expanding section of the population over the next 40 years. Therefore, hypertension prevalence rates are likely to increase further.

Multiple hypertension trials in older populations demonstrated that modest reductions of systolic BP (SBP) resulted in considerable decreases in CVD events [5-9]. The Hypertension in the Very Elderly Trial (HYVET) study showed that antihypertensive therapy in patients aged ≥80 years with the SBP goal of <150 mmHg reduced stroke, cardiovascular events, heart failure, and death [6]. However, the SBP target for older patients varies in different guidelines and the recent Systolic Blood Pressure Intervention Trial (SPRINT) study showed intensive lowering of SBP to an unattended clinic SBP goal <120 mmHg was beneficial in older patients with increased CVD risk but without diabetes [10], including those ambulatory subjects ≥75 years [11].

The SHanghai Elderly Cardiovascular Health Study (SHECHS) was performed to recruit older residents aged ≥65 years living in the Pudong Gaohang Community Medical Center region of Shanghai to provide current data evaluating the prevalence and treatment of hypertension in an older community population.

## MATERIALS AND METHODS

### Study population

The SHECHS is a longitudinal, population-based community study of non-institutionalized adults aged ≥65 years as described previously [12]. All permanent residents aged ≥65 years in the community were invited to participate in the study by local community leaders and poster advertisements. The SHECHS was initiated in 2013 and 3950 participants were recruited with complete baseline data available. Follow-up studies are planned over 5 years including one that has been conducted in 2014.

The study was approved by the institutional review board of Tongji Medical School affiliated Shanghai East Hospital and written informed consent was obtained from each participant before data collection.

### Data collection

The participants who chose to volunteer attended Gaohang community medical center in the morning after overnight fasting for at least 10 hours. After 5 minutes resting, sitting BP was measured twice by trained personnel using a mercury sphygmomanometer and the average of the two BP values was recorded. Medical information including details of medications was obtained by trained family doctors. Anthropometric measurements including body weight, height, and waist circumference were obtained according to a standardized protocol (http://apps.who.int/iris/bitstream/10665/42569/1/9241545763_eng.pdf.). Blood samples were obtained and measured as described previously [12].

### Study outcome definitions

Definite hypertension was defined as an average of two measurements of SBP ≥140 mmHg or diastolic BP (DBP) ≥90 mmHg, or normal BP with concomitant use of antihypertensive medications [13]. Isolated systolic hypertension (ISH) was defined as SBP ≥140 mmHg or normal SBP with concomitant use of antihypertensive medication with normal DBP (<90 mmHg). Definite diabetes mellitus (DM) was defined as fasting serum glucose (FG) ≥7.0 mmol/l or normal FG with concomitant use of insulin or oral hypoglycemic agents. Body mass index (BMI) was defined as weight in kilograms divided by height in meters squared rounded to the nearest 0.1 kg/m^2^. Estimated glomerular filtration rate (eGFR) was calculated by the abbreviated MDRD equation {186 x (creatinine/88.4) - 1.154 x (Age)-0.203 x (0.742 if female)}. Established CVD was defined as history of myocardial infarction (MI), coronary or other arterial revascularization, or stroke confirmed by examination of the medical records.

### 10-year estimated risk of ischemic cardiovascular diseases

As some of the standard CVD risk prediction equations such as the Framingham score have not been found to be accurate in Chinese populations, we used an equation validated by the USA-PRC Collaborative Study and the China Multicenter Collaborative Study of Cardiovascular Epidemiology (China MUCA) Research Group, which used traditional covariates age, SBP, BMI, total cholesterol (TC), DM and smoking to predict the 10-year estimated risk of combined ischemic stroke and coronary events [14]. This equation is heavily weighted by the observed level of BP irrespective of treatment and by the age. As this study assessed an older population, we modified the age score with only 1 additional score per 10 years after age ≥70 years instead of 1 score per 5 years to facilitate looking at the hypertension control details in different CVD risk groups of this older population. The participants were stratified into low (<10%), moderate (10-20%), high (≥20%, or DM), or established CVD) and very high (established CVD with DM) risk groups.

### Statistical analysis

Descriptive statistics were calculated for all variables and prevalence estimates for hypertension and the use of antihypertensive treatments were analyzed separately for participants aged <80 and ≥80 years and according to the 10-year estimated CVD risk group. Significant differences in all continuous categorical variables were determined by Student t-test and percentage values by Chi-squared test (χ2-test). Differences among multiple group variables were determined by ANOVA and two group variables by LSD-t test. All statistical analyses were performed using SPSS17.0 software (SPSS Inc., Chicago, IL, USA). A two-tailed *p* value <0.05 was considered to be statistically significant.

**Table 1:** Demographic and clinical characteristics of subjects stratified by age <80 and ≥80 years

Age (years)	All (n=3950)	<80 (n=3791)	≥80 (n=609)	*P* value
Female, % (n)	55.8 (2205)	55.2 (1843)	59.4 (362)	0.051
Middle school (6yr education), % (n)	58.3 (2301)	64.8 (2166)	22.2 (135)	<0.01
Current cigarette user, % (n)	14.1 (556)	15.0 (502)	8.9 (54)	<0.01
BMI, kg/m^2^	24.6 (24.5-24.7)	24.6 (24.5-24.7)	24.3 (24.0-24.6)	0.014
Established CVD, % (n)	7.8 (307)	7.0 (233)	12.2 (74)	<0.01
Definite hypertension, % (n)	74.8 (2955)	74.1 (2477)	78.5 (478)	0.022
ISH, % (n)	72.7 (2872)	71.9 (2401)	77.3 (471)	0.006
SBP, mmHg	138.8 (138.3-139.3)	138.2 (137.6-138.8)	141.8 (140.5-143.2)	<0.01
DBP, mmHg	81.8 (81.5-82.1)	82.0 (81.7-82.3)	80.9 (80.2-81.6)	0.037
On BP medication, % (n)	67.1 (1984)	67.7 (1676)	64.4 (308)	0.184
Attainment SBP <150 mmHg	62.9 (1247)	63.5 (1065)	59.1 (182)	0.048
Attainment SBP <140 mmHg	37.0 (735)	38.3 (642)	30.2 (93)	<0.01
Definite diabetes, % (n)	20.7 (819)	20.5 (684)	22.2 (135)	0.356
FG, mmol/l	5.7 (5.7-5.8)	5.7 (5.6-5.8)	5.8 (5.7-6.0)	0.106
HbA1c, %	6.3 (6.3-6.4)	6.3 (6.3-6.4)	6.4 (6.3-6.5)	0.029
Hemoglobin, g/L	138.3 (137.8-138.8)	139.0 (138.5-139.5)	134.3 (13.0-135.5)	<0.01
Serum potassium, mmol/L	4.3 (4.3-4.3)	4.3 (4.3-4.3)	4.3 (4.3-4.4)	<0.01
Serum sodium, mmol/L	143.3 (143.2-143.3)	143.3 (143.2-143.4)	143.1 (142.9-143.3)	0.098
Creatinine, μmol/L	76.8 (76.0-77.6)	75.3 (74.6-76.1)	84.8 (82.2-87.4)	<0.01
eGFR, mL/min/1.73 m^2^	78.4 (77.7-79.0)	78.7 (78.0-79.4)	76.7 (75.4-78.0)	0.028
TC, mmol/l	4.9 (4.9-5.0)	4.9 (4.9-5.0)	4.9 (4.8-5.0)	0.475
LDL-C, mmol/l	3.3 (3.2-3.3)	3.3 (3.2-3.3)	3.2 (3.2-3.3)	0.351
HDL-C, mmol/l	1.4 (1.4-1.4)	1.45 (1.4-1.4)	1.5 (1.4-1.5)	<0.01
TG, mmol/l	1.6 (1.5-1.6)	1.6 (1.6-1.7)	1.4 (1.4-1.5)	<0.01
Statin use, % (n)	5.3 (210)	5.2 (173)	6.1 (37)	0.376
Aspirin use, % (n)	14.2 (559)	14.0 (468)	14.9 (91)	0.528

Values are mean and 95% confidence interval (CI), or percentages % (number).

eGFR, estimated glomerular filtration rate using MDRD equation; SBP, systolic blood pressure; DBP, diastolic blood pressure; BMI, body mass index; FG, fasting glucose; TC, total cholesterol; LDL-C, low density lipoprotein cholesterol; HDL-C, high density lipoprotein cholesterol; TG, triglyceride. Definite diabetes mellitus (DM) was defined as fasting serum glucose (FG) ≥7.0 mmol/l or normal FG with concomitant use of insulin or oral hypoglycemic agents.

## RESULTS

### Demographic and clinical characteristics of participants

A total of 3950 participants with mean age 72.0 years, 1745 male and 2205 female, completed the SHECHS baseline examination. There were no significant differences in age, SBP, or prevalence of hypertension, DM and CVD between men and women, as reported previously [12]. Compared to subjects aged <80 years, in those ≥80 years there tended to be a greater proportion of females, the education level was lower, there were fewer cigarette users, and there was lower BMI (Table 1). The older group had more established CVD, higher SBP, lower DBP, and higher prevalence rates of definite hypertension and ISH. In individuals ≥80 years, antihypertensive medication usage tended to be less and a smaller proportion of subjects attained SBP goals <150 mmHg and <140 mmHg compared to the younger group.

**Table 2:** Treatment and control of hypertension according to CVD risk group in Chinese subjects aged ≥65 years

	Low risk	Moderate risk	High risk	Very High risk	*P* value
Total, n=3950					
% (n)	46.3 (1828)	21.9 (866)	28.6 (1131)	3.2 (125)	
SBP, mmHg	131.5 (130.9-132.2)	144.3 (143.3-145.3)^^^^	146.0 (144.9-147.1)^^^^^#^	141.6 (138.5-144.7)^^^^^∆∆^	<0.01
DBP, mmHg	80.0 (79.6-80.4)	83.3 (82.7-83.8)^^^^	83.5 (82.9-84.1)^^^^	82.6 (80.9-84.3)^^^^	<0.01
Definite hypertension, % (n)	62.2 (1137)	86. 4 (748)	84.9 (960)	88.0 (110)	<0.01
On BP medication, % (n)	67.3 (765)	59.4 (444)	70.9 (681)	85.5 (94)	<0.01
Attain SBP <150 mmHg	78.7 (602)	53.2 (236)	52.4 (357)	55.3 (52)	<0.01
Attain SBP <140 mmHg	55.9 (428)	24.1 (107)	25.1 (171)	30.9 (29)	<0.01
TC, mmol/l	4.8 (4.8-4.9)	5.2 (5.1-5.2)^^^^	5.0 (4.9-5.0)^^^^^##^	4.7 (4.5-4.9)^##^	<0.01
LDL-C, mmol/l	3.2 (3.1-3.2)	3.5 (3.4-3.5)^^^^	3.3 (3.2-3.3)^^^^^##^	3.0 (2.9-3.2)^#^^∆∆^	<0.01
Statin treatment, % (n)	2.8 (52)	3.0 (26)	7.5 (85)	37.6 (47)	<0.01
FG, mmol/l	4.9 (4.9-4.9)	5.3 (5.2-5.3)^^^^	7.2 (7.0-7.3)^^^^	7.5 (7.1-7.9)^^^^	<0.01
HbA1c, %	5.8 (5.8-5.8)	6.37 (6.3-6.4)^^^^	7.0 (6.9-7.1)^^^^	7.4 (7.2-7.6)^^^^	<0.01
Aged <80 yr, n=3341					
% (n)	50.1 (1674)	20.1 (671)	27.0 (903)	2.8 (93)	
SBP, mmHg	131.8 (131.1-132.5)	144.5 (143.4-145.7)^^^^	145.2 (144.0-146.5)^^^^	140.2 (136.6-143.9)^^^^^#^^∆∆^	<0.01
DBP, mmHg	80.3 (79.9-80.7)	83.7 (83.1-84.4)^^^^	83.7 (83.1-84.3)^^^^	82.5 (80.65-84.5)^^^	<0.01
Definite hypertension, % (n)	63.2 (1058)	86.1 (578)	84.1 (759)	88.2 (82)	<0.01
On BP medication, % (n)	66.9 (708)	60.6 (350)	71.8 (545)	89.0 (73)	<0.01
Attain SBP <150 mmHg	77.9 (552)	51.7 (181)	53.2 (290)	57.5 (42)	<0.01
Attain SBP <140 mmHg	54.8 (388)	24.0 (84)	26.6 (145)	34.2 (25)	<0.01
Aged =80 yr, n=609					
% (n)	25.3(154)	32.0 (195)	37.4 (228)	5.3 (32)	
SBP, mmHg	128.5 (126.7-130.4)	143.4 (141.6-145.2)^^^^	149.0 (146.6-151.3)^^^^^#^^†^	145.6 (139.9-151.3)^^^^^†^	<0.01
DBP, mmHg	77.1 (75.9-78.3)^†^	81.6 (80.4-82.8)^^^^	82.5 (81.3-83.8)^^^^	82.7 (79.3-86.2)^^^^	<0.01
Definite hypertension, % (n)	51.3 (79)	85.2 (170)	88.2 (201)	87.5 (28)	<0.01
On BP medication, % (n)	72.2 (57)	55.3 (94)	67.7 (136)	75.0 (21)	<0.01
Attain SBP <150 mmHg	87.7 (50)	58.5(55)	49.3 (67)	47.6 (10)	<0.01
Attain SBP <140 mmHg	70.2 (40)	24.5 (23)	19.1 (26)^†^	19.0 (4)	<0.01

SBP, systolic blood pressure; DBP, diastolic blood pressure; FG, fasting glucose; TC, total cholesterol; LDL-C, low density lipoprotein cholesterol.

*p* value is for ANOVA between 4 groups.

^ *p*<0.05,

^^ *p*<0.01, significantly different from low-risk group;

# *p*<0.05,

## *p*<0.01, significantly different from moderate-risk group;

∆∆ *p*<0.01, significantly different from high-risk group;

† *p*<0.05, significantly different from <80 yr.

On BP medication is for hypertensive individuals. Attain SBP goal is for subjects on medication.

**Table 3:** Use of antihypertensive medication in Chinese subjects aged <80 and ≥80 years

Age (years)	Total		<80 years		≥ 80 years		*P* value
With hypertension (n)	2955		2496		470		
On BP medication, % (n)	67.1 (1984)		67.7 (1676)		64.4 (308)		0.184
Attainment SBP <150 mmHg	62.9 (1247)		63.5 (1065)		59.1 (182)		0.048
Attainment SBP <140 mmHg	37.0 (735)		38.3 (642)		30.2 (93)		<0.01
		Multi-meds		Multi-meds		Multi-meds	
CCB, % (n)	54.2 (1075)	42.6 (458)	55.7 (933)	44.2 (412)	46.1 (142)	32.4 (46)	0.002
ARB, % (n)	43.1 (855)	45.1 (386)	44.2 (741)	46.3 (343)	37.0 (114)	37.7 (43)	0.020
ACEI, % (n)	7.1 (140)	47.1 (66)	7.2 (120)	49.2 (59)	6.5 (20)	35.0 (7)	0.809
BB, % (n)	11.0 (218)	74.3 (162)	11.3 (190)	75.8 (144)	9.1 (28)	64.3 (18)	0.276
Diuretic, % (n)	2.6 (52)	73.1 (38)	2.3 (38)	78.9 (30)	4.5 (14)	57.1 (8)	0.031
Chinese compound, % (n)	13.3 (263)	21.7 (57)	12.4 (206)	25.1 (52)	18.2 (56)	8.9 (5)	0.008
1 medication, % (n)	72.4 (1436)		70.9 (1189)		80.2 (247)		0.001
2 medications, % (n)	24.3(482)		25.4 (426)		18.2 (56)		0.006
≥3 medications, % (n)	3.3 (66)		3.6 (61)		1.6 (5)		0.082

% using antihypertensive medications is for individuals who received medications. ACEI, angiotensin converting enzyme inhibitor; ARB, angiotensin receptor blocker; BB, beta-blocker; CCB, calcium channel blocker; SBP, systolic blood pressure.

### Prevalence of hypertension according to cardiovascular disease risk

Moderate CVD risk was present in 21.9% of participants, 28.6% were at high risk and 3.2% at very high risk, and the prevalence of increased CVD risk tended to be greater in subjects aged ≥80 years (Table 2). Compared to low CVD risk subjects, the moderate, high and very high risk individuals had significantly elevated mean SBP and DBP levels, but these values tended to be lower in the very high risk individuals than in high or moderate risk subjects, probably because of higher treatment rates.

The overall prevalence of hypertension was 74.8% and more than 80% of the moderate and higher risk individuals had hypertension with a similar frequency pattern in the two age groups (Table 2). The SBP level of high and very high risk groups in individuals aged ≥80 years was higher than that in younger individuals. In high risk individuals, there was a significantly higher prevalence of hypertension in the group with estimated CVD risk >20% (99.1%) compared to those with DM (81.0%) or established CVD (82.4%) (Supplementary Table 1).

The presence of other CVD risk factors also varied according to the CVD risk level and both FG and HbA1c increased progressively from low to very high risk subjects (Table 2).

### Use of antihypertensive treatment in different cardiovascular disease risk groups and attainment of SBP goals

Overall usage of antihypertensive medication was similar in older and younger individuals but the treatment rate varied between different CVD risk groups with the lowest treatment rate in the moderate risk individuals (Table 2). Older participants tended to have lower rates of treatment than the younger group in the moderate, high and very high CVD risk groups.

The attainment rate of SBP goals in those taking antihypertensive medication varied between different CVD risk groups, probably partly because the recorded level of BP irrespective of treatment is a major determinant of the CVD risk level. The SBP goal of ≤150 mmHg was attained in more low risk individuals than in other risk groups and a similar pattern of attainment of the SBP goal of ≤140 mmHg was observed but the proportions of subjects attaining the lower goals were much less, especially in subjects aged ≥80 years in the high and very high risk groups (Table 2).

### Usage of antihypertensive medications

Antihypertensive medication was used in 67.1% of the subjects with similar usage rates in subjects aged <80 and ≥80 years (Table 1). The most frequently used medications were calcium channel blockers (CCBs) at 54.2%, followed by angiotensin receptor blockers (ARBs) at 43.1%, Chinese fixed-dose combination medications at 13.3%, beta-blockers (BB) at 11.0%, angiotensin converting enzyme inhibitors (ACEIs) at 7.1% and least frequently diuretics at 2.6% (Table 3). The subject ≥80 years old had significantly reduced usage of CCBs and ARBs but increased usage of Chinese fixed-dose combination medications and diuretics (Table 3). There was no difference in the use of ACEIs and BBs in the two age groups.

In subjects taking antihypertensive medication, one medication was used in 72.4%, two medications were used in 24.3% and only 3.3% of subjects used ≥3 medications. Compared to younger subjects aged <80 years, more of the subjects aged ≥80 years used one medication (Table 3), and fewer older individuals used two medications and even fewer older subjects used ≥3 medications. In subjects taking CCB, ARB and ACEI, 42.6%, 45.1% and 47.1% of individuals used these with other antihypertensive medications, while 21.7% of individuals taking Chinese fixed-dose combination medications had additional therapy with other antihypertensive medication, and those taking diuretics and BB had the highest rates of combination therapy at 73.1% and 74.3%, respectively (Table 3). Significantly less combination therapy of all the antihypertensive medications was used in the older compared to the younger subjects (Table 3).

### Antihypertensive medication usage in different CVD risk groups

The usage of CCBs was not significantly different between risk groups but tended to be lower in the subjects aged ≥80 years in the low, moderate and high risk groups compared to those risk groups in younger subjects (Supplementary Table 2). The usage of ARBs was greater in the high and very high risk groups compared to the low and moderate risk groups and tended to be less in the subjects aged ≥80 years compared to the younger subjects in the low, moderate and high risk but not in the very high risk groups (Supplementary Table 2).

Conversely, the fixed-dose combination medications were used less frequently in the high and very high risk groups compared to the low and moderate risk groups and tended to be used more frequently in the subjects aged ≥80 years compared to the younger subjects in the low and moderate risk groups (Supplementary Table 2).

The use of more than one medication tended to increase with increasing risk levels with a similar trend in the younger and older subjects, but the older group tended to have a higher proportion on a single antihypertensive medication in all CVD risk groups. The pattern of medication usage was similar in the three subgroups of the high CVD risk group but there was very high usage of fixed-dose combination medications (22.4%) in the subjects aged ≥80 years with estimated CVD risk >20% (Supplementary Table 3).

## DISCUSSION

Similar to other older populations [15], there was a high prevalence of hypertension of over 80% in moderate, high and very high risk groups, which was predominantly systolic hypertension in the SHECHS community-based participants. Currently, hypertension is present in 46% of patients with known CVD and 72% of those who have suffered a stroke in the U.S [16], and therefore it is imperative to identify elevated BP and implement more effective approaches to achieve optimal control of hypertension in older subjects for reduction of cardiovascular events and mortality. However, achieving success in hypertension control at both the individual patient-level and even more importantly, the population-level, has remained a major challenge [13].

The treatment rates in the SHECHS subjects varied between risk categories from about 60% to 85% and tended to be lower in the older compared to those aged <80 years. Although the overall attainment of the SBP goal <150 mmHg was reasonable at 63.5% in younger subjects and 59.1% in the subjects aged ≥80 years, goal attainment was less satisfactory in moderate, high and very high risk individuals, especially in the subjects aged ≥80 years, partly because the observed level of BP is a major factor in determining the CVD risk status. The low goal attainment rates may also be related to confusion over appropriate targets according to age or CVD risk level from different guidelines. The doctors treating these subjects were unlikely to calculate the 10-year risk score and may not always regard DM as a high CVD risk condition so they may underestimate the CVD risk in many patients.

Optimal management of hypertension in older patients, especially those aged ≥80 years, remains controversial and there is inconsistency for the goal for SBP in older subjects in different guidelines. Most guidelines recommend a general goal of <140/90 mmHg in uncomplicated hypertension [17], and some recommend a goal of <150/90 mmHg for those aged >80 years or with certain other comorbidities [18, 19]. However, the 2014 American guidelines changed the goal BP to <150/90 mmHg for patients aged ≥60 years [20], and the Chinese guidelines for the management of hypertension recommend a goal of <150/90 mmHg in patients aged ≥65 years with uncomplicated hypertension [3]. The SPRINT study showed cardiovascular benefits with intensive lowering of SBP to a goal <120 mmHg compared with the <140 mmHg goal in older patients with increased CVD risk but without DM or prior stroke [10, 11]. The unattended automated office BP measurements in the SPRINT study were made in such a way as to minimize the white-coat effect and are more comparable with self-measured home BP or daytime ambulatory BP than usual clinic BP values [21]. There were more adverse events in the subjects receiving more intensive treatment so these findings should be interpreted with caution [22], but overall these data do support more intensive treatment of hypertension even in patients ≥80 years [11].

There is also controversy over which class of antihypertensive drugs is most appropriate for first line treatment in older patients. The HYVET study showed a benefit with the thiazide-like diuretic indapamide, adding the ACEI perindopril when necessary, but that was compared to placebo rather than other active treatment [6]. The 2013 ESH/ESC guidelines recommend diuretics or CCBs for the treatment of ISH in older patients [19], and American guidelines have favored diuretics [23], whereas the most recent British NICE guidance preferred CCBs [24]. In the present study, CCBs were the most frequently used antihypertensive drugs followed by ARBs, whereas the use of ACEIs and diuretics was uncommon. This probably reflects the opinion of the treating doctors that CCBs and ARBs are effective and well tolerated and that generic drugs from these classes are readily available and reasonably priced. It is generally recognized that Chinese patients develop cough with ACEIs more often than Caucasians [25], and this knowledge may contribute to the low rate of usage of ACEIs. The reason for the low rate of usage of diuretics is not known. It may be that the treating doctors were concerned about electrolyte disturbances with diuretics in these older subjects and they may have considered the CCBs and ARBs would have fewer adverse effects.

Chinese fixed-dose combination medications were used frequently in the subjects aged ≥80 years, particularly those with lower risk, but the usage was less common than in an earlier study of older people in Shanghai where these combination medications were used in 77.6% of hypertensives in a population with a mean age of 68.3 years [4]. There are two commonly used antihypertensive compounds, one containing small doses of reserpine, hydrochlorothiazide, potassium chloride, dihydralazine and some vitamins and the other, Zhenju Jiangyapian antihypertensive tablet, containing small doses of clonidine hydrochloride, hydrochlorothiazide and rutin. These are mentioned in the Chinese hypertension guideline [3], but there is limited published information on their efficacy and a lack of outcome trials. They are probably popular in older patients because of good tolerability and the erroneous perception that they are herbal medicines entirely of natural origin, rather than because of cost issues. Replacement of these fixed-dose combination medications with the more effective combinations of CCBs and ARBs and the addition of diuretics when appropriate might be expected to achieve higher rates of BP control, especially in subjects aged ≥80 years.

This study has several limitations. Firstly, it is a cross sectional observational study in a community-based population and longitudinal data are not yet available. Secondly, the older subjects in the community were invited to participate in the study by local community leaders and poster advertisements and it is likely there is selection bias as subjects with more severe disease or disability might not join the study and we were not able to determine how representative the participants were of the local population. Thirdly, the BP was only measured on one occasion and the average of two values was used to categorize the hypertension status and control and the CVD risk status and there will be some error related to the white-coat effect and lack of repeated measurements.

In conclusion, hypertension was highly prevalent in this older community population and treatment rates were high but goal attainment was low, especially in the moderate, high and very high CVD risk groups. Combinations of effective therapy were used less frequently in subjects aged ≥80 years and compound antihypertensive preparations, which may have limited efficacy, were more often used in this age group than in younger subjects.

## 

**Supplementary Table 1:** Treatment and control of hypertension according to detailed classification of high risk group

		High risk (n=1131)		
	Estimated risk >20% (229)	Diabetes (720)	Established CVD (175)	*P* value
Total, n=1108				
% (n)	20.2 (229)	63.7 (720)	161 (182)	<0.01
SBP, mmHg	164.0 (162.3-165.8)	141.3 (140.1-142.6) ^^^^	141.7 (139.0-144.4) ^^^^	<0.01
DBP, mmHg	89.2 (87.8-90.5)	81.6 (80.9-82.2) ^^^^	83.7 (82.3-85.0) ^^^^ ^#^	<0.01
Definite hypertension, % (n)	99.1 (227)	81.0 (583)	82.4 (150)	<0.01
On BP medication, % (n)	53.7 (123)	61.5 (443)	63.2 (115)	0.073
Attain SBP <150 mmHg	10.6 (13)	59.8 (265)	68.7 (79)	<0.01
Attain SBP <140 mmHg	0 (0)	29.8 (132)	40.0 (39)	<0.01
TC, mmol/l	5.1 (5.0-5.3)	5.0 (4.9-5.1)	4.8 (4.7-5.0) ^^^^ ^#^	0.008
LDL-C, mmol/l	3.5 (3.4-3.6)	3.3 (3.2-3.3) ^^^^	3.2 (3.0-3.3) ^^^^ ^#^	<0.01
Statin treatment, % (n)	2.6 (6)	3.8 (27)	28.6% (52) ^##^	<0.01
Aged <80 yr, n=903				
% (n)	16.7 (151)	67.8 (612)	15.5 (140)	<0.01
SBP, mmHg	163.3 (161.0-165.6)	141.3 (139.9-142.6)	140.9 (137.7-144.1)	<0.01
DBP, mmHg	90.1 (88.5-91.6)	81.8 (81.1-82.6)	84.2 (82.7-85.8)	<0.01
Definite hypertension, % (n)	99.3 (150)	80.6 (493)	82.9 (116)	<0.01
On BP medication, % (n)	49.0 (74)	61.9 (379)	65.7 (92)	0.005
Attain SBP <150 mmHg	5.4 (4)	58.8 (223)	68.5 (63)	<0.01
Attain SBP <140 mmHg	0 (0)	30.1 (114)	33.7 (31)	<0.01
Aged ≥80 yr, n=228				
% (n)	34.2 (78)	47.4 (108)	18.4 (42)	<0.01
SBP, mmHg	159.7 (156.7-162.7)	142.0 (138.7-145.2)	144.9 (139.2-150.6)	<0.01
DBP, mmHg	85.8 (83.6-88.0)	80.0 (78.3-81.7)	82.3 (79.4-85.1)	<0.01
Definite hypertension, % (n)	98.7 (77)	83.3 (90)	81.0 (34)	<0.01
On BP medication, % (n)	62.8 (49)	59.3 (64)	54.8 (23)	0.687
Attain SBP <150 mmHg	18.4 (9)	65.6 (42)	69.6 (16)	<0.01
Attain SBP <140 mmHg	0 (0)	28.1 (18)	34.8 (8)	<0.01

SBP, systolic blood pressure; DBP, diastolic blood pressure; TC, total cholesterol; LDL-C, low density lipoprotein cholesterol.

*p* value is for ANOVA between 4 groups.

^ p<0.05,

^^ p<0.01, significantly different from estimated risk >20% group;

# p<0.05,

## p<0.01, significantly different from diabetes group;

On BP medication is for hypertensive individuals. Attain SBP goal is for subjects on medication.

**Supplementary Table 2:** Antihypertensive medication use in hypertensive subjects according to CVD risk group

	Low risk	Moderate risk	High risk	Very High risk	*P* value
Total, n=1984					
On BP medication, % (n)	41.8 (765)	51.3 (444)	60.2 (681)	75.2 (94)	<0.01
CCB, % (n)	53.7 (411)	51.4 (228)	55.5 (378)	61.7 (58)	0.249
CCB with other meds % (n)	36.5 (150)	42.5 (97)	48.1 (182)	50.0 (29)	
ARB, % (n)	40.0 (306)	40.7 (181)	47.6 (324) ^^^^ ^##^	46.8 (44) ^^^^ ^##^	0.018
ARB with other meds % (n)	39.9 (122)	45.3 (82)	49.1 (159)	52.3 (23)	
ACEI, % (n)	6.5 (50)	6.3 (28)	7.3 (50)	12.8 (12)	0.141
ACEI with other meds % (n)	46.0 (23)	42.9 (12)	50.0 (25)	50.0 (6)	
BB, % (n)	8.9 (68)	13.1 (58)	11.0 (75)	18.1 (17) ^^^	0.017
BB with other meds % (n)	72.1 (49)	62.1 (36)	81.3 (61)	94.1 (16)	
Diuretic, % (n)	2.4 (18)	2.5 (11)	3.1 (21)	2.1 (2)	0.824
Diuret with other meds % (n)	77.8 (14)	72.7 (8)	66.7 (14)		
Chinese medicine, % (n)	14.8 (113)	15.3 (68)	11.6 (79) ^^^ ^#^	3.2 (3) ^^^^ ^##^	0.032
Chmed with other meds % (n)	23.0 (26)	14.7 (10)	24.1 (19)		
1 medication, % (n)	76.1 (582)	74.1 (329)	68.6 (467)	61.7 (58)	0.652
2 medications, % (n)	22.0 (168)	22.5 (100)	27.0 (184)	31.9 (30)	
≥3 medications, % (n)	2.0 (15)	3.4 (15)	4.4 (30)	6.4 (6)	
<80 years, n=1676					
On BP medication, % (n)	42.3 (708)	52.2 (350)	60.4 (545)	78.5 (73)	<0.01
CCB, % (n)	54.4 (385)	53.7 (188)	57.8 (315)	61.6 (45)	0.380
CCB with other meds % (n)	37.7 (145)	45.7 (86)	50.2 (158)	51.1 (23)	
ARB, % (n)	41.0 (290)	43.4 (152)	48.8 (266) ^^^^ ^##^	45.2 (33) ^^^^ ^#^	0.050
ARB with other meds % (n)	40.0 (116)	47.4 (72)	51.1(136)	57.6 (19)	
ACEI, % (n)	6.6 (47)	6.6 (23)	7.2 (39)	15.1 (11)	0.062
ACEI with other meds % (n)	44.7(21)	47.8 (11)	53.8 (21)	54.5(6)	
BB, % (n)	9.0 (64)	13.7 (48)	11.6 (63)	20.54 (15)	0.008
BB with other meds % (n)	73.4 (47)	62.5 (30)	84.1 (53)	93.3 (14)	
Diuretic, % (n)	2.40 (17)	1.71 (6)	2.56 (14)	1.4 (1)	0.794
Diuret with other meds % (n)	76.5 (13)	83.3 (4)	78.6 (11)		
Chinese medicine, % (n)	13.7 (97)	13.1 (46)	11.2 (61)	4.1 (3) ^^^	0.612
Chmed with other meds % (n)	25.8 (25)	28.6 (9)	26.2(16)		
1 medication, % (n)	75.3 (533)	71.4 (250)	66.6 (363)	58.9 (43)	0.570
2 medications, % (n)	22.7 (161)	24.9 (87)	28.1 (153)	34.2 (25)	
≥3 medications, % (n)	2.0 (14)	3.7 (13)	5.3 (29)	6.8 (5)	
≥ 80 years, n=308					
On BP medication, % (n)	37.0 (57)	48.2 (94)	59.6 (136)	65.6 (21)	<0.01
CCB, % (n)	45.6 (26)	42.6 (40)	46.3 (63)	61.9 (13)	0.458
CCB with other meds % (n)	19.2 (5)	27.5 (11)	38.1 (24)	38.5 (5)	
ARB, % (n)	28.1 (16)	30.9 (29)	42.6 (58) ^^^^ ^##^	52.4 (11) ^^^^ ^##^	0.058
ARB with other meds % (n)	37.5 (6)	34.5 (10)	39.7 (23)	36.4 (4)	
ACEI, % (n)	5.3 (3)	5.3 (5)	8.1 (11)	4.8 (1)	0.794
BB, % (n)	7.0 (4)	10.6 (10)	8.8 (12)	9.5 (2)	0.900
Diuretic, % (n)	1.8 (1)	5.3 (5)	5.1 (7)	4.8 (1)	0.737
Chinese medicine, % (n)	28.1 (16)	23.4 (22)	13.2 (18) ^^^^ ^##^	0 (0) ^^^^ ^##^	0.018
Chmed with other meds % (n)	6.3 (1)	4.5 (1)	16.7 (3)		
1 medication, % (n)	86.0 (49)	84.0 (79)	76.5 (104)	71.4 (15)	0.384
2 medications, % (n)	12.3 (7)	13.8 (13)	22.8 (31)	23.8 (5)	
≥3 medications, % (n)	1.8 (1)	2.1 (2)	0.7 (1)	4.8 (1)	

*p* value is for ANOVA between 4 groups.

^ p<0.05,

^^ p<0.01, significantly different from low-risk group;

# p<0.05,

## p<0.01, significantly different from moderate-risk group;

ACEI, angiotensin converting enzyme inhibitor; ARB, angiotensin receptor blocker; BB, beta-blocker; BP, blood pressure; CCB, calcium channel blocker; Diuret, Diuretic; Chmed, Chinese medicine antihypertensive compound.

**Supplementary Table 3:** Antihypertensive medication use in subgroups of the high CVD risk subjects

		High risk (n=1131)		
	Estimated risk >20%	Diabetes	Established CVD	*P* value
Total, n=1131				
% (n)	20.2 (229)	63.66 (720)	16.09 (182)	<0.01
Definite hypertension, % (n)	99.12 (227)	80.97 (583)	82.41 (150)	<0.01
On BP medication, % (n)	53.73 (123)	61.52 (443)	63.18 (115)	0.073
CCB, % (n)	56.09 (69)	54.40 (241)	59.13 (68)	0.654
CCB with other meds % (n)	44.9 (31)	48.5 (117)	50.0 (34)	
ARB, % (n)	40.65 (50)	48.08 (213)	53.04 (61)	0.150
ARB with other meds % (n)	48.0 (24)	48.4 (103)	52.5 (32)	
ACEI, % (n)	5.69 (7)	8.57 (38)	4.34 (5)	0.223
BB, % (n)	8.13 (10)	10.83 (48)	14.78 (17)	0.256
Diuretic, % (n)	6.50 (8)	2.70 (12)	0.89 (1) ^^^	0.032
Chinese medicine, % (n)	16.26 (20)	10.83 (48)	9.56 (11)	0.190
Chmed with other meds % (n)	10.0 (2)	27.1 (13)	36.4 (4)	
1 medication, % (n)	14.60 (71)	11.89 (76) ^^^ ^#^	3.19 (3) ^^^^ ^##^	0.652
2 medications, % (n)	74.07 (360)	68.23 (436)	61.70 (58)	
≥3 medications, % (n)	22.01 (107)	27.69 (177)	31.91 (30)	
< 80 years, n=903				
% (n)	16.72 (151)	67.77 (612)	15.50 (140)	<0.01
Definite hypertension, % (n)	98.71 (77)	83.33 (90)	80.95 (34)	<0.01
On BP medication, % (n)	62.82 (49)	59.25 (64)	54.76 (23)	0.687
CCB, % (n)	64.86 (48)	55.67 (211)	60.86 (56)	0.276
CCB with other meds % (n)	41.7 (20)	51.7 (109)	51.8 (29)	
ARB, % (n)	40.54 (30)	49.34 (187) ^^^^ ^##^	53.26 (49) ^^^^ ^#^	0.247
ARB with other meds % (n)	50.0 (15)	50.3 (94)	55.1 (27)	
ACEI, % (n)	5.40 (4)	8.44 (32)	3.26 (3)	0.184
BB, % (n)	8.10 (6)	10.81 (41)	17.39 (16)	0.127
Diuretic, % (n)	2.70 (2)	2.90 (11)	1.08 (1)	0.612
Chinese medicine, % (n)	12.16 (9)	11.34 (43)	9.78 (9) ^^^	0.877
Chmed with other meds % (n)	11.1 (1)	27.9 (12)	33.3 (3)	
1 medication, % (n)	70.27 (52)	66.75(253)	63.04 (58)	0.570
2 medications, % (n)	25.67 (19)	28.49 (108)	28.26 (26)	
≥3 medications, % (n)	4.05 (3)	4.74 (18)	8.69 (8)	
≥ 80 years, n=228				
% (n)	34.21 (78)	47.36 (108)	18.42 (42)	<0.01
Definite hypertension, % (n)	98.71 (77)	83.33 (90)	80.95 (34)	<0.01
On BP medication, % (n)	62.82 (49)	59.25 (64)	54.76 (23)	0.687
CCB, % (n)	42.85 (21)	46.87 (30)	52.17 (12)	0.755
CCB with other meds % (n)	47.6 (10)	26.7 (8)	41.7 (5)	
ARB, % (n)	40.81 (20)	40.62 (26) ^^^^ ^##^	52.17(12) ^^^^ ^##^	0.598
ARB with other meds % (n)	45.0 (9)	34.6 (9)	41.7 (5)	
ACEI, % (n)	6.12 (3)	9.37 (6)	8.69 (2)	0.815
BB, % (n)	8.16 (4)	10.93 (7)	4.34 (1)	0.620
Diuretic, % (n)	12.24 (6)	1.56 (1)	0 (0)	0.018
Chinese medicine, % (n)	22.44 (11)	7.81 (5)	8.69 (2)	0.059
Chmed with other meds % (n)	18.2 (2)	20 (1)	0	
1 medication, % (n)	69.38 (34)	82.81 (53)	73.91 (17)	0.384
2 medications, % (n)	28.57 (14)	17.18 (11)	26.08 (6)	
≥3 medications, % (n)	2.04 (1)	0 (0)	0 (0)	

BP, blood pressure; ACEI, angiotensin converting enzyme inhibitor; ARB, angiotensin receptor blocker; BB, beta-blocker; CCB, calcium channel blocker.

*p* value is for ANOVA between 3 groups.

^ p<0.05,

^^ p<0.01, significantly different from estimated risk >20% group;

# p<0.05,

## p<0.01, significantly different from diabetes group;

On BP medication is for hypertensive individuals.

ACEI, angiotensin converting enzyme inhibitor; ARB, angiotensin receptor blocker; BB, beta-blocker; BP, blood pressure; CCB, calcium channel blocker; Diuret, Diuretic; Chmed, Chinese medicine antihypertensive compound.
